# Zinc Absorption and Endogenous Fecal Zinc Losses in Bangladeshi Toddlers at Risk for Environmental Enteric Dysfunction

**DOI:** 10.1097/MPG.0000000000002361

**Published:** 2019-04-16

**Authors:** Prasenjit Mondal, Julie M. Long, Jamie E. Westcott, M. Munirul Islam, Mondar Ahmed, Mustafa Mahfuz, Tahmeed Ahmed, Leland V. Miller, Nancy F. Krebs

**Affiliations:** ∗ Nutrition and Clinical Services Division, International Center for Diarrheal Disease Research, Bangladesh (icddr,b), Dhaka, Bangladesh; † University of Colorado School of Medicine, Department of Pediatrics, Section of Nutrition, Aurora, CO.

**Keywords:** inflammation, zinc absorptive capacity, zinc deficiency, zinc homeostasis, zinc intestinal losses

## Abstract

**Objectives:**

Environmental enteric dysfunction (EED) impairs zinc absorption from food, and zinc deficiency may contribute to the poor growth associated with EED. We examined zinc absorption from a standardized aqueous zinc dose, and habitual daily endogenous fecal zinc excretion (EFZ) and compared these outcomes between children grouped by the lactulose to mannitol ratio (L:M).

**Methods:**

Bangladeshi toddlers (18–24 months) with low (<0.09) and high (≥0.09) L:M were administered isotope-labeled 3 mg aqueous zinc in the fasted state. Fractional absorption of zinc (FAZ) and EFZ were measured by dual stable isotope tracer method and an isotope dilution method, respectively. Secondary aims included examining relationships of biomarkers of systemic and intestinal inflammation and gut function with FAZ and EFZ.

**Results:**

Forty children completed the study; nearly all had evidence of EED. No differences in zinc homeostasis measurements (mean ± SD) were observed between high and low L:M groups: FAZ was 0.38 ± 0.19 and 0.31 ± 0.19, respectively; both figures were within estimated reference range. Means of EFZ were 0.73 ± 0.27 and 0.76 ± 0.20 mg/day for high and low L:M, respectively, and were 10% to 15% above estimated reference range. Regression analyses indicated that biomarkers of systemic inflammation were directly associated with increasing FAZ, consistent with increased gut permeability. Biomarkers of intestinal inflammation were negatively associated with EFZ, consistent with low-zinc intake and chronic deficiency.

**Conclusions:**

In these children at risk of EED, endogenous zinc losses were not markedly increased. Results suggest that efforts to improve zinc status in EED should focus on substantially improving zinc intakes.

**What Is Known**

- Environmental enteric dysfunction is heterogeneous and difficult to diagnose.
- Zinc absorption from foods is relatively low in children exhibiting characteristics of environmental enteric dysfunction.
- Endogenous fecal zinc losses have been reported to be positively associated with the lactulose to mannitol urine ratio.

**What Is New**

- In young children with evidence of environmental enteric dysfunction, zinc absorption of an aqueous dose given in fasting state was positively associated with markers of inflammation and gut permeability. Endogenous fecal zinc losses decreased with increasing biomarkers of environmental enteric dysfunction.
- Impaired zinc absorption and low zinc intakes, rather than excessive endogenous zinc losses, may be greater factors predisposing young children with environmental enteric dysfunction to zinc deficiency.

Impaired zinc homeostasis and status may synergistically interact with the complex condition environmental enteric dysfunction (EED). Zinc is critical for maintenance of a normal intestinal barrier, and it has a potent anti-inflammatory function. Both of these roles may be challenged by the pathology of EED. Zinc deficiency has thus been proposed to contribute to the poor growth and morbidity associated with EED (1–3). Zinc homeostasis is controlled through both absorption of dietary zinc and secretion and reabsorption of intestinal endogenous zinc excretion, the latter being most closely associated with chronic zinc status.

Although historically EED has been difficult to diagnose and characterize (4), expert consensus holds that it is a chronic subclinical inflammatory condition of the proximal small intestine resulting in increased permeability and diffuse villous blunting resulting in malabsorption (4–6). With disruptions to the intestinal tract seen with EED, absorption of exogenous zinc and reabsorption of endogenous zinc may be impaired (1).

Few investigations of zinc homeostasis have been undertaken in young children with EED. In 2 reports in 3- to 5-year old children in Malawi, daily absorbed zinc was apparently adequate and was related to dietary zinc intake as expected (7,8), but endogenous fecal zinc (EFZ) excretion was positively associated with the dual sugar urinary lactulose to mannitol ratio (L:M) and contributed to negative zinc balance (7). In contrast, we recently reported that absorption of dietary zinc was lower than predicted over a wide range of zinc intakes in Bangladeshi toddlers with evidence of EED (9). Reported high rates of zinc deficiency (10) and EED (11) in young Bangladeshi children support the need for a better understanding of the effects of marginal dietary zinc intake and EED on zinc homeostasis.

The primary aims of the present study were 2-fold: to measure fractional absorption of zinc (FAZ) from a standardized aqueous zinc dose and to determine the habitual daily EFZ; both measurements were compared between groups of children with normal and abnormal L:M (9,11). A secondary aim was to examine relationships of biomarkers of inflammation and gut function with zinc homeostasis.

## METHODS

### Study Design

This experimental study aimed to measure FAZ of a standard dose of aqueous zinc administered in the postabsorptive state in 18–24-month-old children living in a peri-urban slum area of Dhaka, Bangladesh who were at risk for EED. In the same subjects, habitual daily losses of EFZ were measured while the children consumed a diet similar to their usual intake (Figure, Supplemental Digital Content 1, Study design, clinical protocol and associated outcomes). As previously described, the L:M was used to assign 20 participants per group to high L:M (≥0.09) and low L:M (<0.09) groups (9,11–13). Participants were admitted to the Clinical Trials Unit (CTU) at the International Center for Diarrheal Disease Research, Bangladesh (icddr,b) the evening before initiation of the isotope study procedures. On the day children arrived in the CTU, they also completed procedures for the lactulose to rhamnose urine ratio (L:R), obtained as an additional putative biomarker of EED. To measure FAZ and EFZ, procedures included oral administration of a zinc stable isotope-labeled aqueous dose of zinc; intravenous zinc isotope infusion; measurement of dietary intake; and collections of urine and stool (Figure, Supplemental Digital Content 1). Fractional absorption and EFZ were measured by previously described methods (14–16). Serum and fecal biomarkers of systemic and intestinal inflammation and gut function were measured to further characterize EED. The study was approved by Research Review Committee and Ethical Review Committee of icddr,b as well as the University of Colorado Multiple Institutional Review Board. The study was registered under ClinicalTrials.gov NCT02760095.

### Participants

Participants were recruited and completed the study procedures between October 2016 and April 2017 as previously described for a companion study in children from the same community (9). Inclusion criteria were willingness to comply with the study demands; length-for-age *Z* score (LAZ) between −1.25 and −3.0 (17); and hemoglobin (Hb) ≥8 g/dL. Exclusion criteria included chronic illness; diarrhea treated with zinc supplement within 2 weeks of screening; severe anemia; and severe acute malnutrition.

After written informed consent, 4 days before the isotope studies, the child and guardian returned to the Mirpur health clinic for L:M testing. Field research assistants administered an oral solution of lactulose and mannitol to participants (11); urine was quantitatively collected over the subsequent 2 hours (18). The L:M was used as the primary EED screening tool, and children were grouped by high or low L:M.

### Power and Sample Size

Sample size was calculated to detect a 20% change in EFZ between high L:M and low L:M groups, assuming that the magnitude and variability of the EFZ data would be similar to that observed in a study in rural Malawi (8). Given standard deviation (SD) of 0.2 mg/day and specifying α = 0.5 and power of 0.8, it was determined that 17 children per group would be needed to detect a 0.2 mg/day difference in EFZ between groups.

### Description of Diets: Preparation and Administration

Throughout the stay in the CTU, all meals were prepared on site in the icddr,b food laboratory using standard recipes developed for previous studies to reflect participants’ typical diets (19). For the metabolic period to determine EFZ, pre- and post-weights of all meals were measured, and duplicate diets were collected for analysis of daily total dietary zinc (TDZ) intake.

### Preparation and Administration of Aqueous Zinc (Zn) Dose and Isotopes

Doses of ^67^Zn and ^70^Zn stable isotopes (Trace Science International, Toronto, Canada) were prepared in the Pediatric Nutrition Laboratory at University of Colorado, Denver (UC) following standard methods (15,20). The isotopes were tested for sterility, fungal growth, and pyrogenicity at the beginning of study and at 6-month intervals. Approximately 3 mg of accurately measured aqueous zinc sulfate (American Regent, Shirley, NY) was added directly to accurately measured oral ^70^Zn isotope doses (∼250 μg) before transport to icddr,b.

On Study Day 1, at 07:00–08:00 hours, the oral ^70^Zn-labelled aqueous zinc dose was administered in the fasted state, with no food or liquid (except water) consumed ≥ 2 hours before administration. Approximately 1 hour later, a sterile, accurately measured quantity of ∼400 μg ^67^Zn was intravenously administered (15,20). All dose losses from saliva and blood were collected and analyzed for isotope enrichment to adjust the administered doses.

### Sample Collections

When participants were admitted to the CTU, spot urine and stool samples were collected as baseline specimens before isotope administration; stools were also used for fecal biomarkers of inflammation and permeability. Participants were also administered 1000 mg lactulose and 200 mg l-rhamnose in 10 mL of sterile water (Figure, Supplemental Digital Content 1). Urine was collected for 1 hour immediately following sugar administration for subsequent L:R determination (21).

Three days after isotope administration (Study Days 4–8), complete 24-hour fecal collections were initiated for 4 consecutive days in the CTU. Precautions were taken to avoid cross-contamination of zinc isotope between urine and stool samples, including use of zinc-free urine bags (22). Two spot urine samples (approximately 30 mL at morning and evening) were collected daily. All urine, fecal, and dietary samples were stored at −20 °C until shipment to UC.

On Study Day 8, approximately 5 mL of blood was collected; serum was separated after 30 minutes. Samples were stored at −80 °C at icddr,b until analysis for nutritional and inflammatory biomarkers.

### Sample Analyses

All total zinc and stable isotope enrichment analyses were completed at UC. Urine samples were digested and zinc was separated from other inorganic matter using chelation procedures (15,20). Zinc isotope ratios were measured by inductively coupled plasma mass spectrometry and converted to enrichment (15). Stool samples were ashed, then purified using column chromatography (22) for measurement of the intravenous isotope (^67^Zn) ratios.

Serum inflammatory and nutritional biomarkers were analyzed by the icddr,b Nutrition Biochemistry Lab (9). Fecal inflammatory markers and pathogens, and the L:M and L:R were measured by the icddr,b Parasitology Lab (9,11).

### Data Calculations and Analyses

FAZ was determined using the dual isotope tracer ratio method from urine enrichment of the oral and IV isotopes (14,15). As no data on absorption of a fasting aqueous zinc dose in young children were found in the literature, absorption values were predicted from data on absorption of a similar dose in adults based on 2 different assumptions: that absorption by children and adults will be proportional to small intestine length and to absorption of the same quantity of dietary zinc. In the first calculation an adult FAZ of 0.72 from a 3.2 mg aqueous dose (23) was multiplied by the ratio of estimated small intestine length in children with a height of 77 cm (24,25) and adults (26,27), ie, 0.72 × 360 cm/650 cm, yielding a predicted child FAZ value of 0.40. In the second calculation, the adult fasting FAZ of 0.72 was multiplied by the ratio of the FAZ of 3.2 mg of dietary zinc in children at 20 months (28) and in adults (29), ie, 0.72 × 0.25/0.53, generating a predicted FAZ of 0.34.

Mean EFZ (mg/d) was calculated by dividing the endogenous zinc (total fecal zinc × fecal ^67^Zn enrichment) excreted by the product of average urine ^67^Zn enrichment and the number of collection days (8,16,22). For reference values, we calculated an EFZ of 0.65 mg/day using a published Eq. (30) and also considered a published EFZ value of 0.67 ± 0.23 mg/day observed in toddlers (22).

Composite scores of EED were calculated from the alpha-1 antitrypsin, myeloperoxidase, and neopterin data (31). Summary statistics of FAZ, EFZ, and biomarkers of nutritional status and systemic and intestinal inflammation were calculated for low and high L:M groups, and distributions were examined. Comparisons between groups were performed with the Student's *t*-test or the Mann-Whitney nonparametric test. Associations of FAZ and EFZ with the biomarkers of inflammation and nutritional status were investigated using multiple linear regression analysis. Regression models were evaluated for adherence to regression assumptions and by quality criteria. Data analyses were performed using GraphPad Prism V.7.00 (GraphPad Software, La Jolla, CA) and R statistical software V.3.2.2 (32). A statistical difference or association was defined by a *P* value <0.05. Associations were also evaluated by their impact on the regression model selection criterion (corrected Akaike Information Criterion).

## RESULTS

Forty-six toddlers consented and enrolled in the study with complete sampling and analyses for 40 children. Six participants voluntarily exited the study because parents perceived study demands to be too great or they were no longer interested in study participation. There were no statistical differences for anthropometric and demographic comparisons between groups at baseline with the exception of the L:M, upon which the grouping was based (Table, Supplemental Digital Content 2, Baseline demographic and anthropometric data of Bangladeshi toddlers). The average age of participants was 20 months. Weight and length averaged at 9.2 ± 1.0 kg and 77.2 ± 2.2 cm, respectively, with the average LAZ −2.10 ± 0.43 and average hemoglobin concentration 10.6 ± 1.3 g/dL.

**FIGURE 1:**
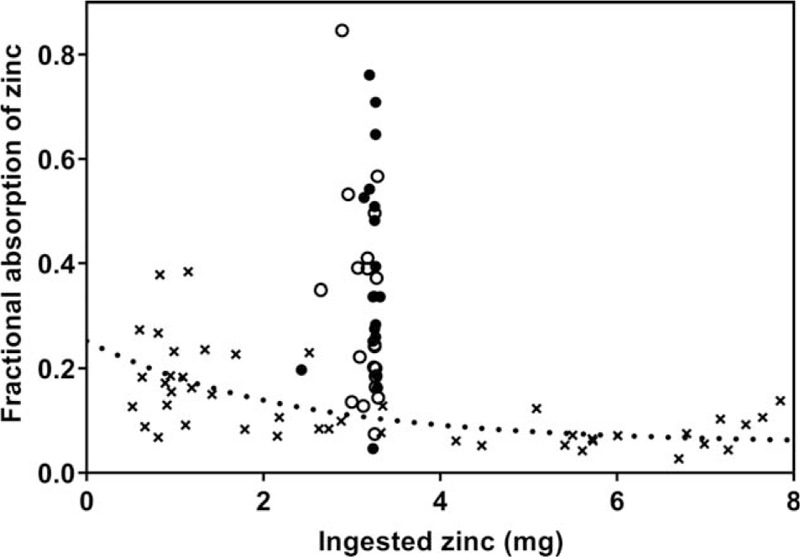
Fractional absorption of zinc versus quantity of ingested zinc shown for the aqueous fasting dose in the present study (circle symbols) and for a zinc-supplemented meal (**x** symbols) administered to a separate group of toddlers from the same Bangladeshi population (9). The greater variability in the fasting FAZ data is apparent, having a standard deviation of 0.19 compared with 0.06 for the FAZ from meals (taking into account the variation in FAZ with quantity of zinc in meal—dotted curve). ●—FAZ from aqueous zinc dose (high L:M, present study); **○**—FAZ from aqueous zinc dose (low L:M, present study); **x**—FAZ from single meal supplemented with zinc. Data from (9). FAZ = fractional absorption of ingested zinc.

Neither of the measures of zinc homeostasis differed statistically between low and high L:M groups (Table 1). The mean (±SD) ingested aqueous doses approximated the planned quantity and did not differ between the groups (3.2 ± 0.2 mg for both, *P* = 0.39). Average FAZs were similar to the predicted values (Table 1), but the variability of the FAZ data was 3 to 4 times larger than that observed for absorption of zinc consumed in meals in other children studied from this same community (9) (Fig. 1). Means for EFZ were also similar between the 2 L:M groups and were 10% to 15% higher than the predictions (Table 1). The TDZ intakes during the 4-day urine and fecal collection period for determination of EFZ were marginally higher for the low L:M group (*P* = 0.10) but were less than the estimated average requirement of 2.5 mg/day for this age (33) for both groups.

Biomarkers of nutritional status and systemic inflammation generally exhibited nonnormal distributions with positive skew, and thus were summarized by median and interquartile range (Table 2). Among these biomarkers, only soluble transferrin receptor (sTfR) and high sensitivity C-reactive protein (hsCRP) showed statistical differences between groups, with the medians for both being higher for the high L:M group. Evidence of iron deficiency was demonstrated by high sTfR and low mean ferritin, also most notable in the high L:M group. Elevated levels were prevalent among all fecal inflammatory biomarkers except myeloperoxidase; none differed by L:M group. The median EED score was slightly higher for the high L:M group: 5 versus 4 for the low L:M group, not a statistical difference.

As the primary outcomes and most secondary outcomes were similar between groups, they were combined and regression analyses were performed on the complete dataset. Regression models demonstrated associations of FAZ and EFZ with multiple biomarkers (Table 3). Fractional absorption was directly associated with tumor necrosis factor-α, hsCRP, and L:R, and it was inversely associated with Hb. EFZ was directly associated with vitamin B_12_ and inversely associated with Hb, neopterin, myeloperoxidase, and L:R. Three dimensional graphs of the modeling of selected covariates exemplify the associations with FAZ and with EFZ (Figure, Supplemental Digital Content 3, 3-dimensional graphs of modeling of selected covariates of FAZ and EFZ). As dietary zinc intake and absorbed zinc are normally associated with EFZ (22,34), TDZ was added as a covariate to examine whether the associations of EFZ with the biomarkers were affected. Controlling for TDZ did not have an appreciable effect on the other relationships in any of the models. Given the expected elevations in biomarker values in the presence of EED, all associations with 1 exception were consistent with increasing FAZ and decreasing EFZ in EED. The sole exception was the inverse relation of EFZ to Hb. No associations of the L:M ratio with either FAZ or EFZ were observed, and the EED score was not found to be related to FAZ.

## DISCUSSION

The major findings from this study of young children with evidence of EED were that neither zinc absorption from a standard dose of aqueous zinc given in the fasting state, nor fecal excretion of endogenous zinc differed between L:M groups. Furthermore, systemic markers of inflammation were positively associated with zinc absorption, whereas markers of intestinal inflammation were inversely related to fecal excretion of endogenous zinc. The L:M did not clearly distinguish EED versus non-EED in that both groups exhibited evidence of systemic and intestinal inflammation, reflecting the heterogeneity of EED and present diagnostic challenges.

Reduced zinc absorption and zinc deficiency have frequently been cited as potential links between EED and the growth failure commonly associated with this condition (1,3,35). Indeed, in a companion study, we reported markedly reduced absorption of zinc from meals in toddlers with evidence of EED (9). The positive association of FAZ with markers of systemic inflammation and intestinal permeability (L:R) observed here appears to contradict the earlier findings, but this is likely a consequence of the different study design. Although average absorption from the fasting dose was similar to that predicted from adult and child reference data, there was a great deal of variability in the FAZ data (with range exceeding an order of magnitude, Fig. 1) compared with either FAZ from meals in children from the same community or from a fasting dose FAZ in adults (23). This suggests that in EED, fasting FAZ of the aqueous dose is influenced to a greater extent by inflammation and permeability, possibly reflecting zinc uptake through nontransporter-mediated mechanisms. We surmise that this would be consistent with the impaired barrier function in the proximal small bowel documented in adults with EED (36,37). These findings may have implications for the efficacy of zinc administered as a liquid supplement or dispersible tablet compared with zinc fortification of foods.

Another factor observed to strongly influence FAZ was Hb concentration, which varied inversely with FAZ. Systemic inflammation, which was evident in many participants, is associated with low Hb, and our observation may reflect the degree of enteropathy.

Contrary to our hypothesis that EFZ would be relatively elevated in these young children, our observed means for the 2 L:M groups were only 10% to 15% above the EFZ predicted by the equation proposed for the European Food Safety Authority (EFSA) (30). Furthermore, the results of our regression modeling indicated that EFZ decreased as biomarkers of intestinal inflammation and permeability increased.

The most plausible explanation for the unexpected findings is chronic zinc deficiency, in which case conservation of endogenous zinc would be the normal homeostatic response. Although data are limited, studies in adults suggest that the site of reabsorption of endogenous zinc is the distal small bowel, and thus may be less impacted by EED compared with absorption of dietary zinc, which occurs primarily in the proximal small bowel where the characteristic pathology of EED is most prominent (3,4,38). The habitual dietary intake of the participants was well below recommended intakes (39), and our findings of impaired zinc absorption from virtually the same diets in similar aged children from the same community resulted in daily absorbed zinc well below estimated physiologic requirements (9). Survey data for children in the communities from which the participants were recruited also indicate high rates of zinc deficiency (10).

Under conditions of normal gut health, EFZ generally varies with habitual daily intake and with absorbed zinc, and it is conserved when intake and absorption are low (22,38–40). In contrast, it has been proposed that young children with EED may experience excessive EFZ losses, representing a disruption of zinc homeostasis and predisposition to zinc deficiency (1,3). This is based in part on observations in other conditions of small bowel pathology with apparently excessive EFZ losses, eg, celiac disease (41). Although data on EFZ directly measured in children with EED are limited, our results contrast with the observations in Malawi of a direct relationship between EFZ and L:M (7).

The strengths of this study include the measurement of FAZ from a standard aqueous dose in fasting state, which allowed us to compare absorptive capacity without the confounding impact of food in the gut, potentially unmasking effects of gut permeability and inflammation. Measurement of EFZ over 4 days in young children with evidence of EED addressed a relative gap in the understanding of the effects of EED on zinc homeostasis. The application of multiple regression modeling to both homeostatic processes provided deeper insights into the influence of multiple covariates of inflammation and nutritional status than is possible from more traditional biomarker measurements.

We also acknowledge limitations of this study. Foremost of these was our inability to distinguish 2 distinct study groups with and without EED based on the L:M screening or subsequently by other potential EED markers. Thus, we did not have a true control group of children from the same environment and with similar diets. Our findings do, however, characterize relationships across a range of biomarkers. Determination of EFZ based on the isotope dilution method is dependent on complete fecal collections, and it is possible that the collections were incomplete, which would lead to underestimates of EFZ. However, the absolute EFZ quantity may be less important than the associations identified by the regression analyses.

The findings from this study confirm that the alterations in small bowel integrity associated with EED are likely to impact zinc homeostasis. Gut permeability and systemic inflammation were associated with enhanced absorption of an aqueous dose of elemental zinc, which contrasts to observations of impaired zinc absorption from food across a wide range of intakes in young children with EED (9). Distinct from others’ findings in older children, we did not observe dramatically increased EFZ losses in our population. We tentatively conclude that in the typical context of EED, the combination of low zinc intakes and compromised zinc absorption contribute more toward suboptimal zinc status than increased losses. Efforts to improve zinc status should thus focus on substantially increasing zinc intakes.

## Supplementary Material

Supplemental Digital Content

## Figures and Tables

**TABLE 1:** Fractional absorption of ingested zinc from standardized aqueous zinc dose in fasting state; daily endogenous fecal zinc, and total dietary zinc by lactulose to mannitol ratio (L:M) group^∗^ during the 4-day metabolic period

	Estimated reference values^†^	High L:M (n = 20)	Low L:M (n = 20)	*P*
FAZ	(0.34–0.4)	0.38 ± 0.19	0.31 ± 0.19	0.25
EFZ, mg/day	(0.65–0.67)	0.73 ± 0.27	0.76 ± 0.20	0.67
TDZ, mg/day		1.78 ± 0.93	2.26 ± 0.85	0.10

Values presented as mean±SD. EFZ = endogenous fecal zinc; FAZ = fractional absorption of zinc; TDZ = total dietary zinc.

^*^High L:M ≥0.09, low L:M <0.09.

^†^Estimated reference values from previously reported data on adults and children (see text).

**TABLE 2:** Biochemical data of Bangladeshi toddlers by lactulose to mannitol ratio (L:M) groups^∗^

	Normal range^†^	high L:M (n = 18–20)	low L:M (n = 17–20)	*P*^‡^
Serum biomarkers of nutritional status			
Zinc, mg/L	0.65–1.18	0.83 (0.73, 1.35)	1.02 (0.74, 1.48)	0.61
Zinc, μmol/L	9.9–18.1	12.7 (11.2, 20.7)	15.6 (11.3, 22.6)	
Ferritin, ng/mL	20–200	15 (7.6, 22)	22 (9.6, 37)	0.27
Ferritin, pmol/L	44.9–449	33.7 (17.1, 49.4)	49.4 (21.6, 83.1)	
Soluble transferrin receptor, μg/mL	2.2–6.3	7.7 (5.8, 13)	5.0 (3.9, 8.7)	0.04
Soluble transferrin receptor, nmol/L	25.9–74.1	90.6 (68.2, 153)	58.8 (45.9, 102)	
Vitamin B12, pg/ML	264–1215	307 (222, 475)	364 (291, 496)	0.46
Vitamin B12, pmol/L	195–897	227 (164, 351)	269 (215, 366)	
Retinol, μg/dL	≥20	33 (26, 39)	33 (28, 38)	0.95
Retinol, μmol/L	0.70	1.15 (0.91, 1.36)	1.15 (0.98, 1.33)	
Serum biomarkers of inflammation			
α-1 acid glycoprotein, mg/dL	50–120	98 (84, 120)	83 (66, 108)	0.09
High sensitivity C-reactive protein, mg/L	0.1–2.8	2.0 (0.48, 5.8)	0.41 (0.14, 1.07)	0.005
Tumor necrosis factor-α, pg/mL	<29.4	31 (29, 34)	29 (27, 32)	0.08
Urine and fecal biomarkers of intestinal function and inflammation			
Lactulose to rhamnose ratio	n/a	0.16 (0.08, 0.23)	0.09 (0.04, 0.15)	0.10
% lactulose excretion, L:M	n/a	0.20 (0.10, 0.49)	0.12 (0.06, 0.19)	0.04
% lactulose excretion, L:R	n/a	0.20 (0.10, 0.38)	0.10 (0.0.04, 0.17)	0.02
Calprotectin, μg/g	<50	115 (34, 337)	64 (33, 110)	0.21
Myeloperoxidase, ng/g	<2000	1161 (421, 3383)	668 (144, 2635)	0.70
Neopterin, nmol/L	<70	1026 (668, 1593)	954 (535, 1945)	0.67
α-1-antitrypsin, mg/g	<0.27	0.40 (0.15, 0.79)	0.17 (0.12, 0.35)	0.14

Values are presented as median (interquartile values).

^*^High L:M ≥ 0.09, low L:M < 0.09.

^†^References for normal ranges provided in previous publication (9).

^‡^Mann-Whitney nonparametric test.

**TABLE 3:** Regression models of fractional absorption of zinc and endogenous fecal zinc for Bangladeshi toddlers

Covariate	Parameter estimates	*P*
FAZ Model (*R*^2^ = 0.52)		
Hemoglobin	−0.077	0.0006
Tumor necrosis factor-α	0.017	0.029
High sensitivity C-reactive protein	0.011	0.041
Lactulose to rhamnose ratio	0.43	0.035
EFZ Model (*R*^2^ = 0.66)
Hemoglobin	−0.095	0.0002
Vitamin B_12_	0.00049	0.0020
Myeloperoxidase^*^	−0.000028	0.065
Neopterin^*^	−0.000055	0.094
Lactulose to rhamnose ratio	−0.54	0.019
FAZ	−0.43	0.021

EFZ, endogenous fecal zinc; FAZ, fractional absorption of zinc.

^*^If either myeloperoxidase or neopterin are removed from this model, *P* for the other drops to <0.004.
