# Supplementary material for: Zinc Absorption and Endogenous Fecal Zinc Losses in Bangladeshi Toddlers at Risk for Environmental Enteric Dysfunction
Source: J Pediatr Gastroenterol Nutr. 2019 Apr 16;68(6):874–9. doi: 10.1097/MPG.0000000000002361 (PMC6553983; doi:10.1097/MPG.0000000000002361)
Supplement: Supplemental Digital Content [file jpga-68-874-s001.pdf]

Figure, Supplemental Digital Content 1.

Study design, clinical protocol, and associated outcomes.

	Study Day	Procedure	Associated Outcome
**Baseline Period**	-4<br><br>0	• L:M screening for group assignment<br>• Baseline urine & fecal collections<br>• L:R assessment	
**Isotope & Zinc Dosing Day**	1	• Oral aqueous Zn + ^{70}Zn<br>• IV ^{67}Zn	
**Metabolic Period**	4<br>5<br>6<br>7	• Partial urine collections (^{70}Zn, ^{67}Zn)<br>• Complete fecal + partial urine collections (^{67}Zn)	Fractional absorption of aqueous Zn<br>Endogenous fecal Zn excretion
	8	• Blood draw	Biomarkers: Inflammation & nutritional status

IV = intravenous; L:M = lactulose to mannitol ratio; L:R = lactulose to rhamnose ratio; Zn = zinc

Table, Supplemental Digital Content 2. Baseline demographic and anthropometric data of Bangladeshi toddlers by lactulose to mannitol ratio (L:M) group[a]

	high L:M (n=20)	low L:M (n=20)	*P*
L:M	0.26±0.21	0.04±0.02	<*0.0001*
Age, *mo*	20±2	20±1	1.00
Gender, n, M/F	12/8	7/13	
Length, *cm*	77.3±2.4	77.0±2.0	0.67
Weight, *kg*	9.3±0.93	9.1±1.1	0.54
LAZ	-2.14±0.38	-2.06±0.47	0.56
WAZ	-1.60±0.64	-1.56±0.91	0.87
WLZ	-0.76±0.91	-0.84±1.14	0.81
Hb, *g/dL*	10.6±1.4	10.5±1.2	0.81

Values are presented as mean±SD unless otherwise noted.

[a]high L:M ≥ 0.09, low L:M <0.09.

F = female; Hb = hemoglobin; LAZ = length-for-age Z-score; M = male; WAZ = weight-for-age Z-score; WLZ = weight-for-length Z-score.

Figure, Supplemental Digital Content 3.

Three-dimensional graphs of modeling of selected covariates of fractional absorption of zinc and endogenous fecal zinc to exemplify associations in Bangladeshi toddlers.

A

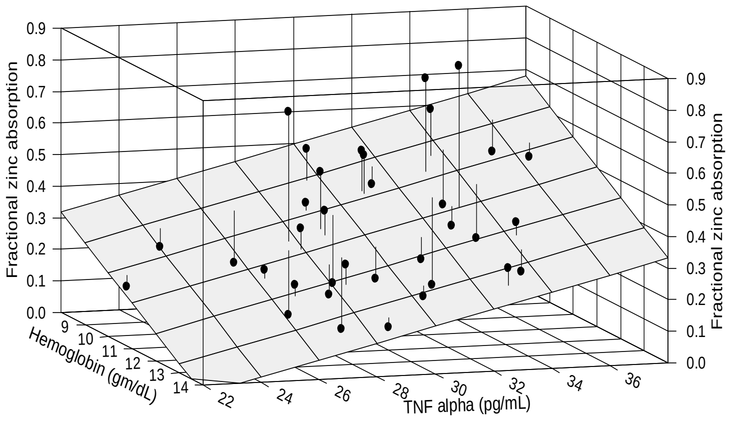

B

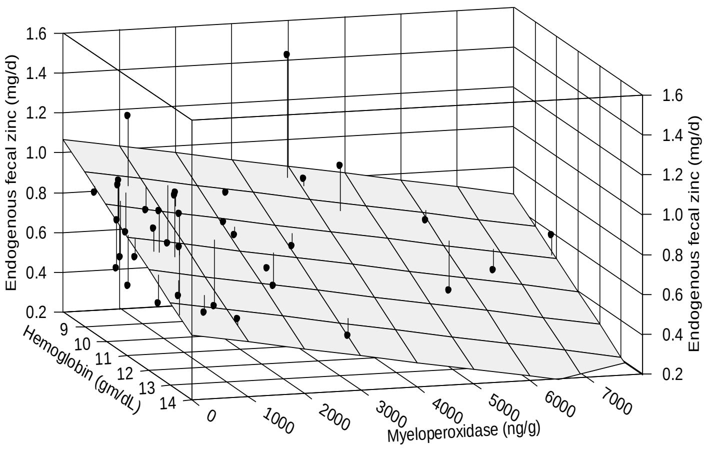

Gray surface shows model having least squares fit to data for the pairs of covariates and response variables. The signs and magnitudes of the slopes of the relationships are evident, and similar to those of the models with additional covariates (Table 3). The vertical lines from data symbols to the model surface show the deviations of the data from the model.
